# 1519. Prevalence and factors associated with HIV testing among sexually experienced high school students in the U.S.A.

**DOI:** 10.1093/ofid/ofad500.1354

**Published:** 2023-11-27

**Authors:** Geoffrey K Kangogo

**Affiliations:** Saint Louis University, Saint Louis, Missouri

## Abstract

**Background:**

HIV testing is a critical strategy for the prevention of HIV/AIDS yet testing among sexually experienced adolescents remains low. This study examined the prevalence of HIV testing and its influential factors among high school students in the USA.

**Methods:**

We analyzed cross-sectional data from the 2019 National Youth Risk Behavior Survey. Analysis was limited to 3123 complete cases of sexually experienced high school students as measured by ever having sex. Chi-square and binary logistic regression analyses involved three models comprising predisposing, enabling and need factors were conducted to study lifetime HIV testing.

**Results:**

The prevalence of lifetime HIV testing was 18.8% with females getting tested more than males. Female gender (OR:1.55; p< 0.001), being Black (OR:1.42; p=0.008), and sexual debut below 13 years (OR:1.67; p=0.013) were positively associated with lifetime HIV testing. Further, we observed that having multiple sexual partners (OR:3.09; p< 0.001) and mild physical activity (OR:1.28; p=0.025) were significantly associated with HIV testing. Lastly, we noted that Hispanics/Latinos (OR:0.67; p=0.025) and those using condoms (OR:0.77; p=0.015) were less likely to have reported ever having an HIV test.

**Conclusion:**

Our study revealed that HIV testing among sexually experienced high school students remains low and sexual risk behaviors that impede testing among students need to be addressed to effectively scale up testing for this vulnerable population. The implication of this study to the relevant stakeholders responsible for creating a healthier nation is to strengthen the current efforts targeting sexually experienced high school students to encourage HIV tests and linkage to care.

**Disclosures:**

**All Authors**: No reported disclosures

